# Roles of orexinergic and noradrenergic neuronal activity in ketamine-induced analgesia: A study using an orexin/ataxin-3 transgenic rat model

**DOI:** 10.1016/j.jatmed.2024.10.003

**Published:** 2024-11-26

**Authors:** Mitsuru Tonosaki, Tetsuya Kushikata, Yoshikazu Nikaido, Daiki Takekawa, Hirotaka Kinoshita, Jyunichi Saito, Kazuyoshi Hirota

**Affiliations:** a Intensive Care Unit, Hirosaki University Hospital, Honcho 53, Hirosaki 0368563, Japan; b Department of Anesthesiology, Hirosaki University Graduate School of Medicine, Zaifu 5, Hirosaki 0368562, Japan; c Department of Metabolomics Innovation, Hirosaki University Graduate School of Medicine, Zaifu 5, Hirosaki 0368562, Japan; d Department of Anesthesiology, Hirosaki University Hospital, Honcho 53, Hirosaki 0368563, Japan

**Keywords:** Ketamine, Orexin, Noradrenaline, Supraspinal acute analgesia, *N*-(2-Chloroethyl)-*N*-ethyl-2-bromobenzylamine hydrochloride

## Abstract

**Background:**

Orexin, a neuropeptide, physiologically interacts with locus coeruleus noradrenergic (LC-NA) activity. We hypothesized that this interaction affects ketamine-induced acute analgesia.

**Methods:**

We assessed ketamine-induced acute analgesia in transgenic (TG) and wild-type (WT) rats with genetically degraded orexinergic neurons using hotplate and tail-flick tests. We examined the effect of the LC-NA toxin, *N*-(2-Chloroethyl)-*N*-ethyl-2-bromobenzylamine hydrochloride (DSP4) on ketamine-induced acute analgesia in separate WT and TG groups. Rats received intra-peritoneal ketamine and intracerebroventricular (ICV) treatments, including saline, orexin, orexin + SB-334867 (an orexin type-1 receptor antagonist), or SB-334867. We measured orexin and noradrenaline levels in the brain regions of untreated WT and TG rats (n = 7 and 5, respectively).

**Results:**

TG exhibited a decreased percentage of maximum possible effect area under the curve (AUC) for hotplate latency (WT: 1315.0 ± 1026.0; TG: 439.6 ± 831.5; P = 0.016) but not tail-flick latency (WT: 1203.0 ± 332.4; TG: 1127.0 ± 512.4; P = 0.732). Orexin (10 nmol, ICV) increased hotplate latency AUC in DSP4-free WT (control: 220.2 ± 227.2; orexin 10: 1575.0 ± 760.2; P = 0.004), DSP4-free TG (control: 119.9 ± 197.1; orexin 10: 866.5 ± 213.2; P = 0.001), DSP4-treated WT (control: −20.0 ± 350.5; orexin 10: 775.4 ± 485.2; P = 0.048), and DSP4-treated TG (control: −181.3 ± 272.6; orexin: 1038.0 ± 652.6; P = 0.040) rats. AUC with orexin 10 was lower in DSP4-treated WT rats than in DSP4-free WT rats (1575.0 ± 760.2 vs. 775.4 ± 485.2; P = 0.025). Co-administration of SB-334867 (20 nmol, ICV) with orexin blocked the effect of orexin on hotplate latency in DSP4-free but not DSP4-treated rats. TG rats had lower noradrenaline contents (pg/mg tissue) in the pons (WT: 341.9 ± 36.3; TG: 279.6 ± 54.4; P=0.038) and cerebral cortex (WT: 1195.1 ± 36.1; TG: 120.1 ± 32.6; P=0.042), but not in the hypothalamus (WT: 895.6 ± 130.4; TG 822.3 ± 1140.3; P=0.373). Orexin levels decreased in all brain regions of TG rats (pons: WT: 7.6 ± 3.2; TG: 1.1 ± 0.3, P = 0.001; hypothalamus: WT: 12.6 ± 2.9; TG: 2.8 ± 1.4, P<0.0001; cerebral cortex: WT: 2.1 ± 0.6; TG: 0.7 ± 0.3, P = 0.001).

**Conclusions:**

The role of orexin in ketamine-induced acute analgesia partly depends on LC-NA activity.

## Introduction

Orexin is a wakefulness-promoting substance, and it has been shown to have analgesic effects as well.^1^, ^2^ Orexinergic neurons innervate the locus coeruleus noradrenergic (LC-NA) neurons,^3^ and intra-cerebroventricular (ICV) injection of orexin activates LC-NA.^4^ Activation of LC-NA activity facilitates analgesia;^5^, ^6^ thus, orexin may exert its analgesic effect by interacting with LC-NA.

Ketamine-induced acute analgesia involves the activation of the descending antinociceptive^7^ and LC-NA^8^ systems. However, when LC-NA is selectively destroyed by *N*-(2-Chloroethyl)-*N*-ethyl-2-bromobenzylamine hydrochloride (DSP4), the analgesic effect of ketamine on thermal nociception is attenuated.^9^ Ketamine also affects orexin activity in the brain,^10^ suggesting that its analgesic mechanism may involve orexin and LC-NA.

However, the exact mechanism of action of orexin on ketamine-induced acute analgesia and the impact of orexin or noradrenaline in this mechanism remains unknown. We hypothesized that orexinergic neurons affect the analgesic effect of ketamine by interacting with LC-NA. We conducted studies using transgenic rats with a destroyed orexin system and wild-type rats to elucidate the relationship between orexin, LC-NA activity, and thermal nociception in ketamine-induced acute analgesia.

To assess the orexin and noradrenergic activity in wild-type and orexin/ataxin-3 transgenic rats with genetically deactivated orexinergic neurons, we decapitated untreated rats and measured their orexin and noradrenaline levels. Measurements were taken from the pons (including the locus coeruleus), hypothalamus (containing orexin cells), and cerebral cortex (receiving noradrenergic projections from the locus coeruleus only). To compare the analgesic effects of ketamine on thermal nociceptive stimuli in wild-type and transgenic rats, we used hot plate and tail-flick tests, which reflect supra-spinal and spinal analgesic processes, respectively.

To determine the effects of orexin and LC-NA on ketamine-induced acute analgesia, we tested orexin (ICV) and/or SB-334867, an orexin type-1 receptor antagonist, on hotplate latency with ketamine in separate wild-type and transgenic rat groups, with or without treatment using DSP4, a selective LC-NA neurotoxin.

## Materials and methods

The Institutional Committee on Animal Research of Hirosaki University Graduate School of Medicine approved this study. Following the ARRIVE guidelines, we used male orexin/ataxin-3 transgenic (weight = 375.4 ± 45.3 g, n = 92) and wild-type (weight = 349.8 ± 46.3 g, n = 94) rats.

### Orexin/Ataxin-3 transgenic rats

The orexin/ataxin-3 transgene expresses an N-terminally truncated human ataxin-3 protein with a Q77-polyglutamine stretch controlled by the human prepro-orexin promoter.^11^, ^12^ Heterozygous transgenic rats were provided by Professor Masashi Yanagisawa (International Institute for Integrative Sleep Medicine, University of Tsukuba, Japan). We used in-house-bred 8–16-week-old first-generation heterozygous transgenic rats and 7–16-week-old wild-type littermate rats. Previous studies have shown that by 4 weeks of age, orexin-expressing cells in transgenic rats are significantly reduced in the perifornical region of the lateral hypothalamus, with further declines at 7, 10, and 13 weeks of age, while wild-type animals maintained a normal distribution of orexin neurons at 4 and 17 weeks.^11^

### Experimental protocol

Rats were housed in a 12-h light/dark cycle environment (lights on 08:00–20:00) at 23.0 ± 1.0 °C. The animals were provided with food and water ad libitum, except on the day of the experiment. All experiments were conducted between 12:00 and 17:00 to minimize the effects of diurnal rhythm on analgesia.

### Ketamine-induced acute analgesia study

Although orexin deficiency could potentially affect pain test outcomes, no abnormal behaviors were observed during the pain test.

### Effects of ketamine on hotplate latency in transgenic and wild-type rats

Each rat (transgenic and wild-type; n = 15 each) was individually placed on a hotplate (MK-350D; Muromachi Kikai Co, Ltd., Tokyo) set at 50 °C, and the reaction time was recorded from the moment when the rat was placed on the plate until hind paw licking occurred. The cutoff latency was set at 60 s to avoid tissue damage.^13^ Two pre-injection values were recorded for each rat, followed by six values measured every 10 min after receiving ketamine (15 mg/kg, IP), as described in previous studies.^7^, ^9^ The percentage of maximum possible effect (% MPE) for hotplate reaction time was calculated using the following formula: % MPE = (posttreatment latency – pretreatment latency)/(cutoff latency-pretreatment latency) × 100.^14^ We calculated the net % MPE using GraphPad Prism version 6.01 for Windows (GraphPad Software, La Jolla, CA, USA).

### Effects of ketamine on tail-flick latency in the transgenic and wild-type rats

Sixteen rats were used in this study. Each rat was individually placed on a tail-flick apparatus (MK-330BTM; Muromachi Kikai Co., Ltd., Tokyo), where radiant heat was applied to the tail, and the reaction time from the onset to the withdrawal of the tail was measured. The heat intensity was adjusted such that the baseline latencies were between 2 and 6 s. A cutoff latency of 10 s was used to avoid tissue damage. Two successive values were measured as pre-injection values, and six successive values were measured every 10 min for each animal after the intraperitoneal injection of ketamine 15/mg (n = 8 each). The injections and % MPE calculations were performed simultaneously with the hotplate test.

### Effects of orexin and SB-334867 on ketamine hotplate latency in transgenic and wild-type rats with or without DSP4 treatment

**Fig. 1:**
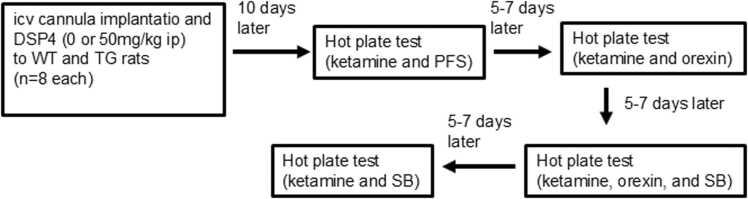
Effects of orexin and SB-334867 on ketamine hotplate latency in transgenic and wild-type rats with or without DSP4 treatment. Rats received either 0 or 50 mg/kg DSP4 (Sigma-Aldrich, St. Louis, MO, USA) intraperitoneally. After a 10-day recovery period, each rat repeatedly received 5–7-day interval ketamine (15 mg/kg, IP) and one of the following intra cerebro-ventricular (ICV) treatments: pyrogen-free saline (PFS), 10 nmol orexin (American Peptide, Sunnyvale, CA, USA), 10 nmol orexin + 20 nmol SB-334867, an orexin type-1 receptor antagonist (SB; Abcam, Cambridge, UK), and 20 nmol SB-334867 alone. Orexin and SB-334867 were dissolved in PFS. Subsequently, hotplate latency was measured.

An ICV cannula was implanted in the lateral cerebral ventricle of each rat with medetomidine hydrochloride (0.15 mg/kg) + midazolam (2 mg/kg) + butorphanol tartrate (2.5 mg/kg) intraperitoneally and penicillin G (5.0 mg/kg) intramuscularly. Simultaneously, rats received either 0 or 50 mg/kg DSP4 (Sigma-Aldrich, St. Louis, MO, USA) intraperitoneally. After a 10-day recovery period, each rat repeatedly received 5–7-day interval ketamine (15 mg/kg, IP) and one of the following ICV treatments: pyrogen-free saline (PFS), 10 nmol orexin (American Peptide, Sunnyvale, CA, USA), 10 nmol orexin + 20 nmol SB-334867, an orexin type-1 receptor antagonist (SB; Abcam, Cambridge, UK), and 20 nmol SB-334867 alone. Orexin and SB-334867 were dissolved in PFS. Subsequently, hotplate latency was measured (Fig. 1).

### Measurement of noradrenaline and orexin content in the central nervous system

The pons, hypothalamus, and cerebral cortex were collected from untreated control rats (wild-type, n = 7; transgenic, n = 5). Rats used for noradrenaline and orexin quantification studies were decapitated, and their brains were rapidly removed. These brain regions were dissected from the surrounding structures and immediately sonicated in a physiological saline solution. The supernatant was collected after centrifugation and stored at −70 °C until enzyme-linked immunosorbent assay (ELISA) or noradrenaline measurement was performed. Commercial ELISA kits (Peninsula Laboratories International, San Carlos, CA, USA) were used to quantify orexin levels, with intra- and inter-assay variation coefficients of 5 %.

Noradrenaline levels were measured directly by high-performance liquid chromatography (ESA Coulochem Model 5100 A, Tokyo, Japan) using a C18 reverse-phase column (4.6 × l50 mm, MC Medical, Tokyo, Japan). The mobile phase buffer contained NaH_2_PO_4_ (0.05 M), CCl_3_COOH (0.05 M), CH_3_(CH_2_)_11_OSO_3_Na (0.7 mM), EDTA_2_Na (0.02 mM), acetonitrile, and methanol at a ratio of 85:10:5, adjusted to pH 3.4, and maintained at 40 °C with a flow rate of 1 mL/min. Noradrenaline was quantified using an electrochemical detector at 300 mV (optimum voltage for oxidation). The lower detection limit was 15 pg/mL, and the intra-assay coefficient of variation was 3.3 %.

### Statistical analysis

Data are expressed as means ± standard deviations (SDs). The area under the curve (AUC) of the % MPE from 10 to 60 min after intraperitoneal ketamine administration was calculated (cumulative AUC). Statistical analyses included the unpaired t-test to compare orexin and noradrenaline concentrations, as well as the cumulative AUC, between wild-type and transgenic rats. Analysis of variance (ANOVA) followed by Šídák's multiple comparisons test was used to assess time-dependent changes in % MPE for the hotplate and tail-flick tests, while ANOVA followed by Tukey's multiple comparisons test was applied to evaluate the cumulative AUC of % MPE among transgenic and wild-type rats with or without DSP4. All statistical analyses were performed using GraphPad Prism version 9.1.2 Windows (GraphPad Software). Statistical significance was set at P < 0.05.

### Sample size calculation

We investigated the effect of ketamine (15 mg/kg, IP) on hotplate latency with or without DSP4.^9^ Based on previous studies, we estimated a minimal sample size of eight per group to achieve a power (1 − β error probability) of 0.8, α error probability of 0.05, and effect size of 1.60 using G*Power 3.1.9.7 (Heinrich Heine University Düsseldorf, Düsseldorf, Germany).

## Results

### Ketamine-induced acute analgesia study

#### Effects of ketamine on hot plate latency in transgenic and wild-type rats

**Fig. 2:**
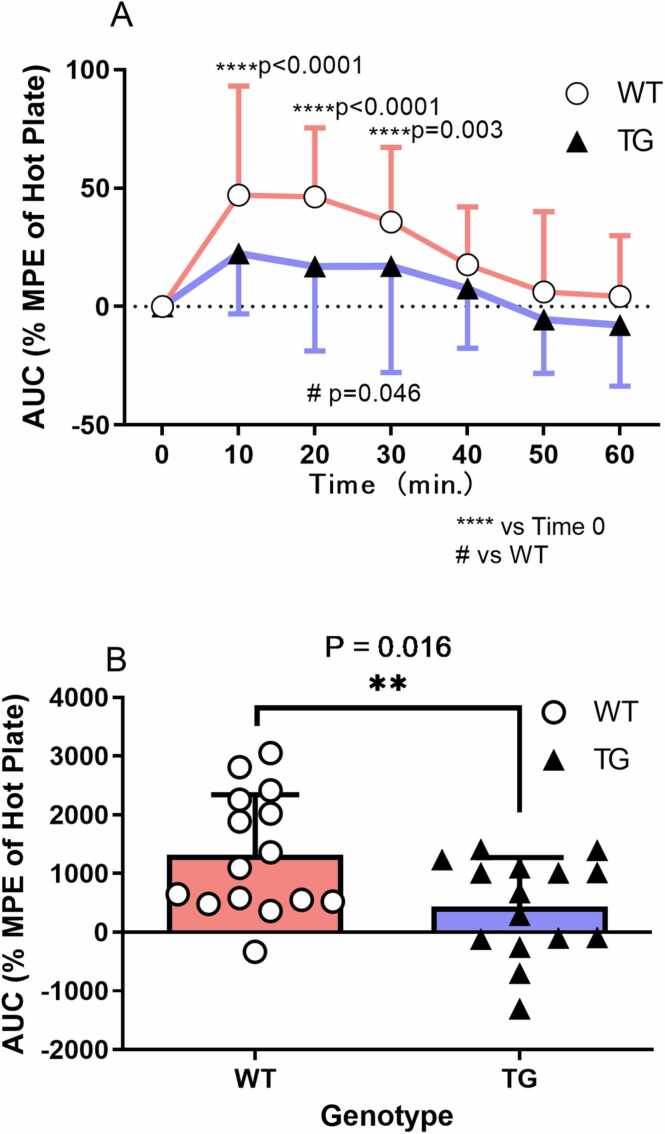
Effects of ketamine on hot plate latency in transgenic and wild-type rats. Intraperitoneal ketamine (15 mg/kg) significantly prolonged hot plate latency (% MPE) in wild-type rats: 43.8 ± 46.0 at 10 min, 43.5 ± 27.9 at 20 min, 31.7 ± 28.4 at 30 min, whereas no prolongation was observed in transgenic ratsrepeated measures two-way ANOVA: genotype, F_1,28_ = 8.59, P = 0.007; time, F_6,168_ = 9.82, P < 0.0001; genotype × time, F_6,168_ = 0.94, P = 0.465, A). The wild-type rats exhibited a greater area under the curve, which was calculated from 10 min to 60 min after ketamine administration (wild-type, 1315.0 ± 1026.0; transgenic, 439.6 ± 831.5; t = 2.567, df = 28, P = 0.016, B).

Before ketamine administration, hot plate latency was 24.9 ± 6.7 s and 22.1 ± 5.1 s in wild-type and transgenic rats, respectively, with no statistically significant difference (t = 1.280, df = 28, P = 0.211). In wild-type rats, ketamine (15 mg/kg, IP) significantly increased the hot plate latency (% MPE) to 43.8 ± 46.0 at 10 min, 43.5 ± 27.9 at 20 min, and 31.7 ± 28.4 at 30 min. However, ketamine had no significant effect on % MPE in transgenic rats (Fig. 2A; repeated measures two-way ANOVA: genotype, F_1,28_ = 8.59, P = 0.0067; time, F_6,168_ = 9.82, P < 0.0001; genotype × time, F_6,168_ = 0.94, P = 0.465). The cumulative AUC of % MPE from 10 to 60 min after ketamine administration was significantly higher in wild-type rats (wild-type, 1315.0 ± 1026.0; transgenic, 439.6 ± 831.5; t = 2.567, df = 28, P = 0.016; Fig. 2B).

#### Effects of ketamine on tail-flick latency in transgenic and wild-type rats

**Fig. 3:**
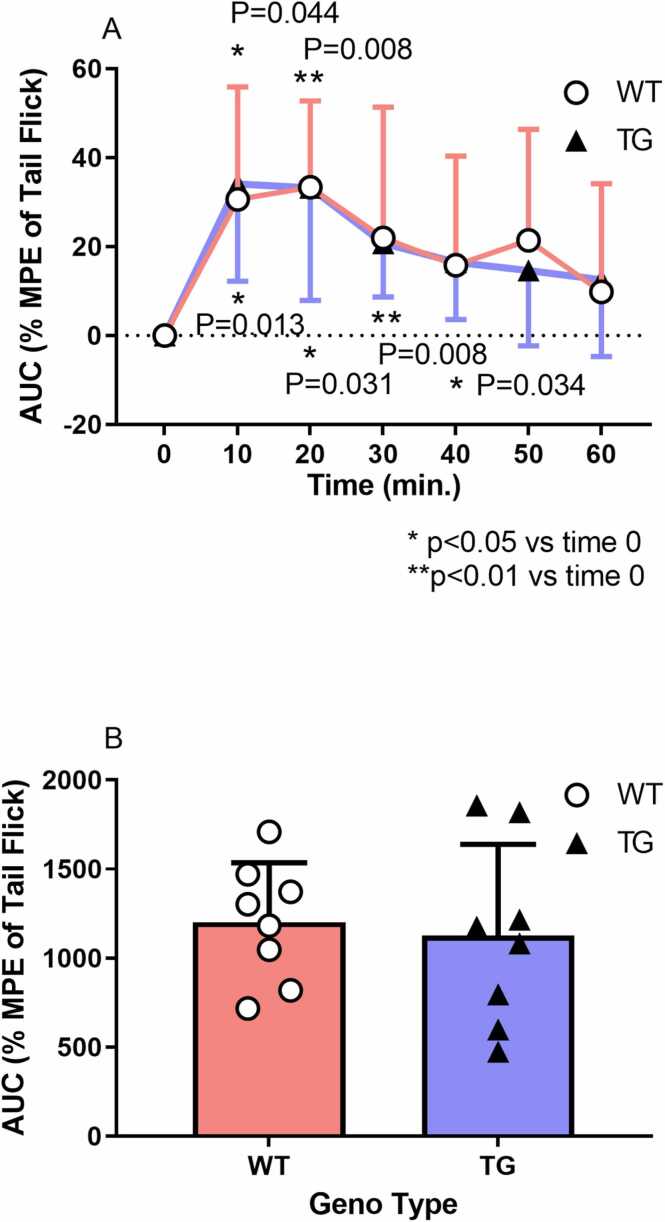
Effects of ketamine on tail-flick latency in transgenic and wild-type rats. Intraperitoneal ketamine (15 mg/kg) significantly prolonged tail-flick latency (% MPE) in wild-type (330.6 ± 25.3 at 10 min, 33.4 ± 19.4 at 20 min) and transgenic (34.1 ± 21.9 at 10, 33.2 ± 25.3 at 20, 20.8 ± 12.1 at 30 min, and 16.5 ± 112.9 at 40 min) rats (repeated measures two-way ANOVA: genotype, F_1,14_ = 1.43, P = 0. 252; time, F_6,84_ = 6.021, P < 0.0001; genotype × time, F_6,84_ = 0.2001, P = 0.976; A). The cumulative area under the curve for tail-flick latency was not significantly different between transgenic and wild-type rats (wild-type, 1203.0 ± 332.4; transgenic, 1127.0 ± 512.4; t = 0.3499, df = 14, *P* = 0.732; B).

Before ketamine administration, tail-flick latency was 4.0 ± 0.8 s and 3.6 ± 0.6 s in wild-type and transgenic rats, respectively, with no statistically significant difference (t = 1.265, df = 14, P *=* 0.223). Ketamine (15 mg/kg, IP) significantly prolonged tail-flick latency (% MPE) in wild-type (330.6 ± 25.3 at 10 min, 33.4 ± 19.4 at 20 min) and transgenic (34.1 ± 21.9 at 10 min, 33.2 ± 25.3 at 20 min, 20.8 ± 12.1 at 30 min, 16.5 ± 112.9 at 40 min) rats (repeated measures two-way ANOVA: genotype, F_1,14_ = 1.43, P = 0.252; time, F_6,84_ = 6.021, P *<* 0.0001; genotype × time, F_6,84_ = 0.2001, P = 0.976; Fig. 3A). The cumulative AUC for tail-flick latency showed no significant difference between wild-type (1203.0 ± 332.4) and transgenic rats (1127.0 ± 512.4; t = 0.3499, df = 14, P = 0.732; Fig. 3B)*.*

#### Effect of orexin and SB-334867 on ketamine hotplate latency in transgenic and wild-type rats with or without DSP4 treatment

**Fig. 4:**
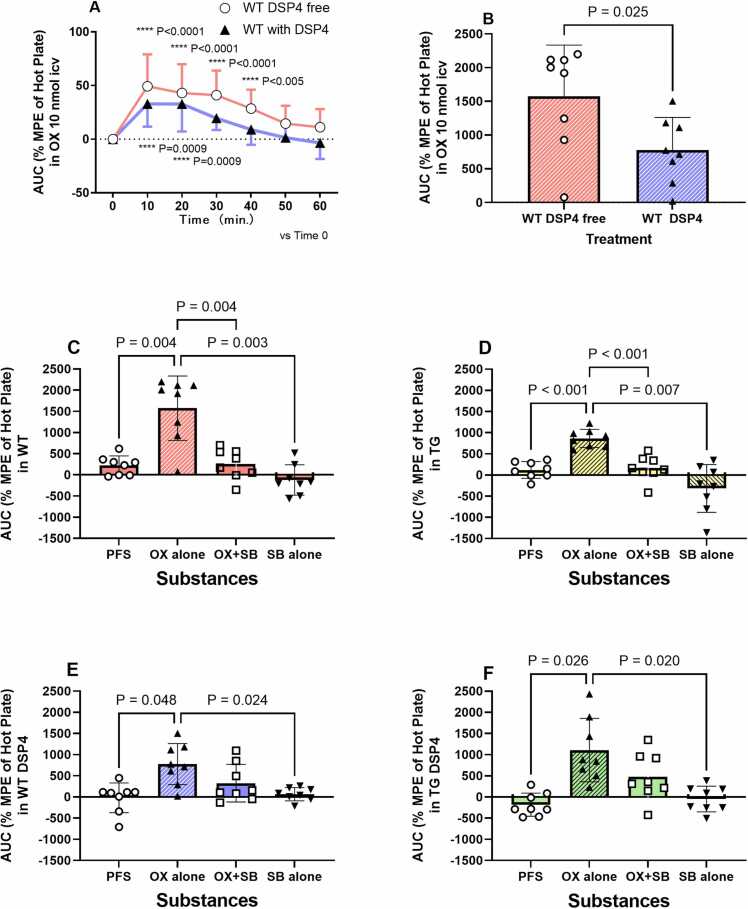
Orexin (10 nmol, ICV) with ketamine (15 mg/kg) significantly prolonged the hotplate latency (% MPE) in DSP4-free (49.3 ± 29.7 at 10 min, 43.0 ± 26.9 at 20 min, 41.1 ± 22.9 at 30 min, and 28.5 ± 17.7 at 40 min) and DSP4-treated wild-type (32.9 ± 21.3 at 10 min and 32.8 ± 25.6 at 20 min) rats (repeated measures two-way ANOVA: genotype, F_1,14_ = 6.45, P = 0.023; time, F_6, 84_ = 16.42, P < 0.0001; genotype × time, F_6, 84_ = 0.74, P = 0.618; A). Orexin (10 nmol) increased the cumulative area under the curve of hotplate latency in DSP4-free wild-type rats compared to DSP4-treated wild-type rats (wild-type, 1575.0 ± 760.2; transgenic, 775.4 ± 485.2; t = 2.506, df = 14, P = 0.025; B). Orexin (10 nmol, ICV) increased the cumulative area under the curve of hotplate latency in DSP4-free wild-type (control: 220.2 ± 227.2; orexin 10: 1575.0 ± 760.2, P = 0.004; C), DSP4-free transgenic (control: 119.9 ± 197.1; orexin 10: 866.5 ± 213.2, P = 0.001; D), DSP4-treated wild-type (control: −20.0 ± 350.5; orexin 10: 775.4 ± 485.2, P = 0.048; E), and DSP4-treated transgenic (control: −181.3 ± 272.6; orexin: 1108.0 ± 749.7, P = 0.026; F) rats. SB-334867 (20 nmol) co-administered with orexin (10 nmol) antagonized the orexin effect in DSP4-free wild-type (OX 10: 1575.0 ± 760.2; co-administration: 256.3 ± 353.2, P＝0.004; C) and in DSP4-free transgenic rats (OX 10: 866.5 ± 213.2; co-administration: 163.9 ± 294.9, P＝0.0002; D). SB-334867 (20 nmol), administered alone, also antagonized the orexin effect in DSP4-free wild-type (OX 10: 1575.0 ± 760.2; SB-334867 alone: 120.6 ± 357.6, P＝0.003; C), DSP4-free transgenic (OX 10: 866.5 ± 213.2; SB-334867 alone: −318.0 ± 565.1, P＝0.007; D), DSP4-treated wild-type (OX 10: 775.4 ± 485.2; SB-334867 alone: −65.7 ± 156.2, P＝0.023; E), and DSP4-treated transgenic (OX 10: 1108.0 ± 749.7; SB-334867 alone: −48.6 ± 302.6, P＝0.020; F) rats.

Orexin (10 nmol, ICV) significantly prolonged hot plate latency (% MPE) with ketamine (15 mg/kg) in DSP4-free (49.3 ± 29.7 at 10 min, 43.0 ± 26.9 at 20 min, 41.1 ± 22.9 at 30 min, and 28.5 ± 17.7 at 40 min) and DSP4-treated (32.9 ± 21.3 at 10 min and 32.8 ± 25.6 at 20 min) wild-type rats (repeated measures two-way ANOVA: genotype, F_1,14_ = 6.45, P = 0.023; time, F_6,__84_ = 16.42, P < 0.0001; genotype × time, F_6,__84_ = 0.74, P = 0.618; Fig. 4A). Orexin increased the cumulative AUC of hotplate latency more in DSP4-free wild-type rats than in DSP4-treated wild-type rats (wild-type, 1575.0 ± 760.2; transgenic, 775.4 ± 485.2; t = 2.506, df = 14, P = 0.025; Fig. 4B). Orexin (10 nmol, ICV) significantly increased the cumulative AUC of hotplate latency in DSP4-free wild-type (control: 220.2 ± 227.2; orexin 10: 1575.0 ± 760.2, P = 0.004; Fig. 4C), DSP4-free transgenic (control: 119.9 ± 197.1; orexin 10:866.5 ± 213.2, P = 0.001; Fig. 4D), DSP4-treated wild-type (control: −20.0 ± 350.5; orexin 10: 775.4 ± 485.2, P = 0.048; Fig. 4E), and DSP4-treated transgenic (control: −181.3 ± 272.6; orexin: 1038.0 ± 652.6, P = 0.020; Fig. 4F) rats. SB-334867 (20 nmol, ICV) + orexin (10 nmol) reversed the orexin-induced enhancement of ketamine analgesia in DSP4-free wild-type (orexin 10: 1575.0 ± 760.2; co-administration: 256.3 ± 353.2, P＝0.004 vs. orexin 10; Fig. 4C) and DSP4-free transgenic (orexin 10: 866.5 ± 213.2; co-administration: 163.9 ± 294.9, P＝0.0002 vs. orexin 10; Fig. 4D) rats.

In addition, sole administration of SB-334867 (20 nmol, ICV) antagonized the orexin effect in DSP4-free wild-type (orexin 10: 1575.0 ± 760.2; SB-334867 alone: 120.6 ± 357.6, P＝0.003 vs. orexin 10; Fig. 4B), DSP4-free transgenic (orexin 10: 866.5 ± 213.2; SB-334867 alone: −318.0 ± 565.1, P＝0.007 vs. orexin 10; Fig. 4C), DSP4-treated wild-type (orexin 10: 775.4 ± 485.2; SB-334867 alone: −65.7 ± 156.2, P＝0.024 vs. orexin 10; Fig. 4D), and DSP4-treated transgenic (orexin 10: 1099.0 ± 762.1; SB-334867 alone: −48.6 ± 302.6, P＝0.022 vs. orexin 10; Fig. 4E) rats.

#### Measurement of noradrenaline and orexin content in the central nervous system

Wild-type rats had higher noradrenaline and orexin levels than transgenic rats in all three regions of the brain: the pons, hypothalamus, and cerebral cortex (Table 1).

**Table 1:** Inter-group comparison of noradrenaline and orexin levels in untreated wild-type and transgenic rats.

	**Noradrenaline content (pg/mg wet tissue) in wild rats**				
**Regions**	**Genotype**				**P*-*value**
	**Wild-type**	**n**	**Transgenic**	**n**	
Pons	341.9±36.3	7	279.6±54.4	5	0.0377
Hypothalamus	895.6±130.4	7	822.3±140.3	5	0.3733
Cerebral Cortex	195.1±36.1	7	120.1±32.6	5	0.0042
	**Orexin content (pg/mg wet tissue) in wild rats**				
**Regions**	**Genotype**				**P-value**
	**Wild-type**	**n**	**Transgenic**	**n**	
Pons	7.6±3.2	7	1.1±0.3	5	0.0012
Hypothalamus	12.6±2.8	7	2.9±1.4	5	<0.0001
Cerebral Cortex	2.1±0.6	7	0.7±0.3	5	0.0013

## Discussion

We confirmed that the noradrenaline and orexin levels in the pons, hypothalamus, and cerebral cortex were lower in the transgenic rats. Noradrenergic neurons are divided into two major systems: one originating from the locus coeruleus and the other from the ventral brainstem. Noradrenergic neurons in the cerebral cortex are only innervated by the locus coeruleus. However, those in the hypothalamus are solely innervated by the extra-locus coeruleus and the ventral brainstem. The pons has a locus coeruleus. Comparing the noradrenaline levels in these three regions helps determine whether noradrenergic neurons from the locus coeruleus are selectively disrupted.^15^ A previous study demonstrated that orexinergic mutation affects noradrenaline levels in the cerebral cortex of canine.^16^ This study supports our findings that noradrenaline levels were different between wild-type and transgenic rats. We chose the DSP-4 dose based on previous studies^9^, ^17^, ^18^ indicating that DSP-4 (50 mg/kg) reduced noradrenaline levels in the locus coeruleus innervated region such as cerebral cortex, hippocampus, and pons by approximately 70 %–80 %. The selective disruption of noradrenergic neurons in the locus coeruleus attenuates ketamine-induced acute analgesia.^9^ Orexinergic neurons originate from the hypothalamus, and orexin excites locus coeruleus noradrenergic neurons. Therefore, we selected these sites to study the interactions between noradrenaline and orexin in ketamine-induced acute analgesia. Untreated wild-type rats, but not transgenic ones, showed an increased cumulative AUC for hot plate latency after ketamine administration.

Hot plate latency is used to evaluate supra-spinal analgesia.^19^ Orexin administered ICV prolongs hot plate latency via type 1 receptors,^20^ and ketamine activates orexin neurons^21^; hence, increased orexin activity may contribute to the acute analgesic effects of ketamine. Orexin also activates LC-NA via the orexin type 1 receptor,^22^ influencing the supra-spinal analgesic effects of ketamine.^9^ Therefore, orexin could modulate ketamine analgesia through its interaction with LC-NA activity. Notably, the cumulative AUC of ketamine-induced hotplate latency decreased in DSP4-free transgenic and DSP4-treated wild-type and transgenic rats. Orexin (10 nmol, ICV) increased the cumulative AUC, while LC-NA deactivation by DSP4 inhibited it in wild-type rats. Orexin promoted ketamine analgesia; however, co-administration with SB-334867 inhibited it in DSP4-free wild-type and transgenic rats. These findings indicate that orexin influences ketamine analgesia via the orexin type 1 receptor, suggesting that orexin may potentiate the supra-spinal analgesic effect of ketamine by interacting with LC-NA activity.

In this study, we found that orexin prolonged the hotplate latency of ketamine in wild-type and transgenic rats. The effect of orexin was antagonized by SB-334867 (20 nmol, ICV). These findings indicate that orexinergic activation contributes to ketamine-induced acute analgesia through orexin type1 receptors. However, ketamine increased the cumulative AUC of tail-flick latency in both wild-type and transgenic rats. The tail-flick latency reflects the spinal analgesic process.^19^ The results suggest that orexin does not mediate the analgesic effects of ketamine on spinal thermal nociception.

This study had some limitations. While we explored the roles of orexin and noradrenaline in ketamine-induced acute analgesia, we could not exclude interactions with other neurotransmitters, such as acetylcholine, histamine, serotonin, and gamma-aminobutyric acid,^23^, ^24^ which are involved in hypnosis and analgesia.^21^ Additionally, differences in sleep-wake patterns between transgenic and wild-type rats may influence ketamine-induced acute analgesia. Although we focused on the relationship between noradrenaline and ketamine in this study, other aspects of this relationship warrant further investigation. We recommend analyzing data for males and females separately to prevent sex bias and enhance the generalizability of findings, keeping in mind the potential effects of the estrous cycle in females. The estrous cycle affects orexin dynamics^25^ and analgesia^26^; therefore, it is essential to align female data with this cycle. Although rodent estrous cycles last 3–4 days, obtaining data on the same estrous cycle at our facility is difficult. Although the data were limited to males, we reported the findings related to acute analgesia induced by orexin, noradrenaline, and ketamine. Data from females remain valuable for further studies. The tail-flick results suggest that ketamine may be involved in the spinal analgesic process. However, further research is needed to clarify this mechanism.

## Conclusions

Orexinergic neuronal activity is involved in the supraspinal analgesic effects of ketamine in response to thermal stimulation. The role of orexin in ketamine-induced acute-analgesia partly depends on LC-NA activity. These findings suggest that targeting orexin receptors could enhance the analgesic effects of ketamine. Further research is required to explore the therapeutic potential of orexin receptor modulators combined with ketamine for pain management in clinical settings.

## CRediT authorship contribution statement

**Hirotaka Kinoshita:** Writing – review & editing, Project administration, Data curation, Conceptualization. **Jyunichi Saito:** Writing – review & editing, Project administration, Data curation. **Kazuyoshi Hirota:** Writing – review & editing, Supervision, Data curation, Conceptualization. **Mitsuru Tonosaki:** Writing – original draft, Investigation, Data curation, Conceptualization. **Tetsuya Kushikata:** Writing – review & editing, Funding acquisition, Data curation, Conceptualization. **Yoshikazu Nikaido:** Writing – review & editing, Project administration, Methodology, Formal analysis. **Daiki Takekawa:** Writing – review & editing, Project administration, Methodology, Data curation. All authors read and approved the final version of the manuscript.

## Disclosure statement

Not applicable.

## Ethical statement

This study followed the ARRIVE guidelines and was approved by the Institutional Committee on Animal Research of Hirosaki University Graduate School of Medicine (protocol code AE-01-2022-021 and 3 March 2022).

## Funding

This work was supported by the Ministry of Education, Culture, Sports, Science, and Technology of Japan [grant numbers 20K09236, 18K08807, and 26462327].

## Declaration of competing interest

The authors declare that they have no known competing financial interests or personal relationships that could have appeared to influence the work reported in this paper.

Kazuyoshi Hirota is an Editorial Board Member for Journal of Anesthesia and Translational Medicine and was not involved in the editorial review or the decision to publish this article.

## Data Availability

The datasets used and analyzed during the current study are available from the corresponding author upon reasonable request.
